# Supplementary material for: Multiple Nucleocapsid Structural Forms of Shrimp White Spot Syndrome Virus Suggests a Novel Viral Morphogenetic Pathway
Source: Int J Mol Sci. 2023 Apr 19;24(8):7525. doi: 10.3390/ijms24087525 (PMC10140842; doi:10.3390/ijms24087525)
Supplement: Supplementary file 1 [file ijms-24-07525-s001.zip › ijms-2304625-supplementary.pdf]

SUPPLEMENTARY MATERIAL

Multiple nucleocapsid structural forms of shrimp white spot syndrome virus suggests a novel viral morphogenetic pathway

Hui-Ju Huang[1,2†], Sen-Lin Tang[3†], Yuan-Chih Chang[2], Hao-Ching Wang [4,5,6], Tze Hann Ng [4,7,8], Rees F. Garmann[9], Yu-Wen Chen[3], Jiun-Yan Huang[4], Ramya Kumar[4,7], Sheng-Hsiung Chang[4,7], Shang-Rung Wu[10], Chih-Yu Chao[11,12], Kyoko Matoba[13], Iwasaki Kenji[14], William M. Gelbart[15], Tzu-Ping Ko[2], Hwei-Jiung (Andrew) Wang[2,5], Chu-Fang Lo[4,7], Li-Li Chen[*,1,16], Han-Ching Wang[*, 4,7]

[1] Institute of Marine Biology, National Taiwan Ocean University, Keelung 20224, Taiwan

[2] Institute of Biological Chemistry, Academia Sinica, Nankang, Taipei 115, Taiwan

[3] Biodiversity Research Center, Academia Sinica, Taipei 11529, Taiwan

[4] International Center for the Scientific Development of Shrimp Aquaculture, National Cheng Kung University, Tainan 701, Taiwan

[5] The Ph.D. Program for Translational Medicine, College of Medical Science and Technology, Taipei Medical University and Academia Sinica, Taipei 110, Taiwan

[6] Graduate Institute of Translational Medicine, College of Medical Science and Technology, Taipei Medical University, Taipei 110, Taiwan

[7] Department of Biotechnology and Bioindustry Sciences, National Cheng Kung University, Tainan 701, Taiwan

[8] Temasek Life Sciences Laboratory, National University of Singapore, Singapore 117604

[9] Department of Chemistry and Biochemistry, San Diego State University, San Diego, California 92182-1030, USA

[10] Institute of Oral Medicine, National Cheng Kung University, Tainan 701, Taiwan

[11] Department of Physics and Graduate Institute of Applied Physics, National Taiwan University, Taipei 10617, Taiwan

[12] Molecular Imaging Center, National Taiwan University, Taipei 10617, Taiwan

[13] Laboratory for Protein Synthesis and Expression, Institute for Protein Research, Osaka University, Osaka 565-0871, Japan

[14] Life Science Center for Survival Dynamics, Tsukuba Advanced Research Alliance (TARA), University of Tsukuba, Tsukuba 305-8577, Japan

[15] Department of Chemistry and Biochemistry, University of California, Los Angeles, California 90095-1569, USA

[16] Center of Excellence for the Oceans, National Taiwan Ocean University, Keelung 20224, Taiwan

[†] These authors contributed equally to this work

*Co-corresponding authors: Prof. Li-Li Chen (email: joechen@mail.ntou.edu.tw), Prof. Han-Ching Wang (email: wanghc@mail.ncku.edu.tw)

Figure S1. (A) Schematic diagram of the length and width of WSSV intact virion and nucleocapsid, based on imagine with TEM and cryo-EM. To quantify the observation for TEM with UA staining, the length (longest dimension) and width (shortest dimension) of ~100 particles of each type were measured and plotted as a length distribution and number of segments per nucleocapsid histogram. For cryo-EM, ~20 particles of each type were measured. Intact enveloped virions as well as the nucleocapsid inside were shorter and fatter than naked nucleocapsids. Average length and width of both intact-enveloped virions (indicated by black letters) and nucleocapsids under cryo-EM were greater than measurements under TEM. (B) Distribution of number of segments assembling the nucleocapsid.

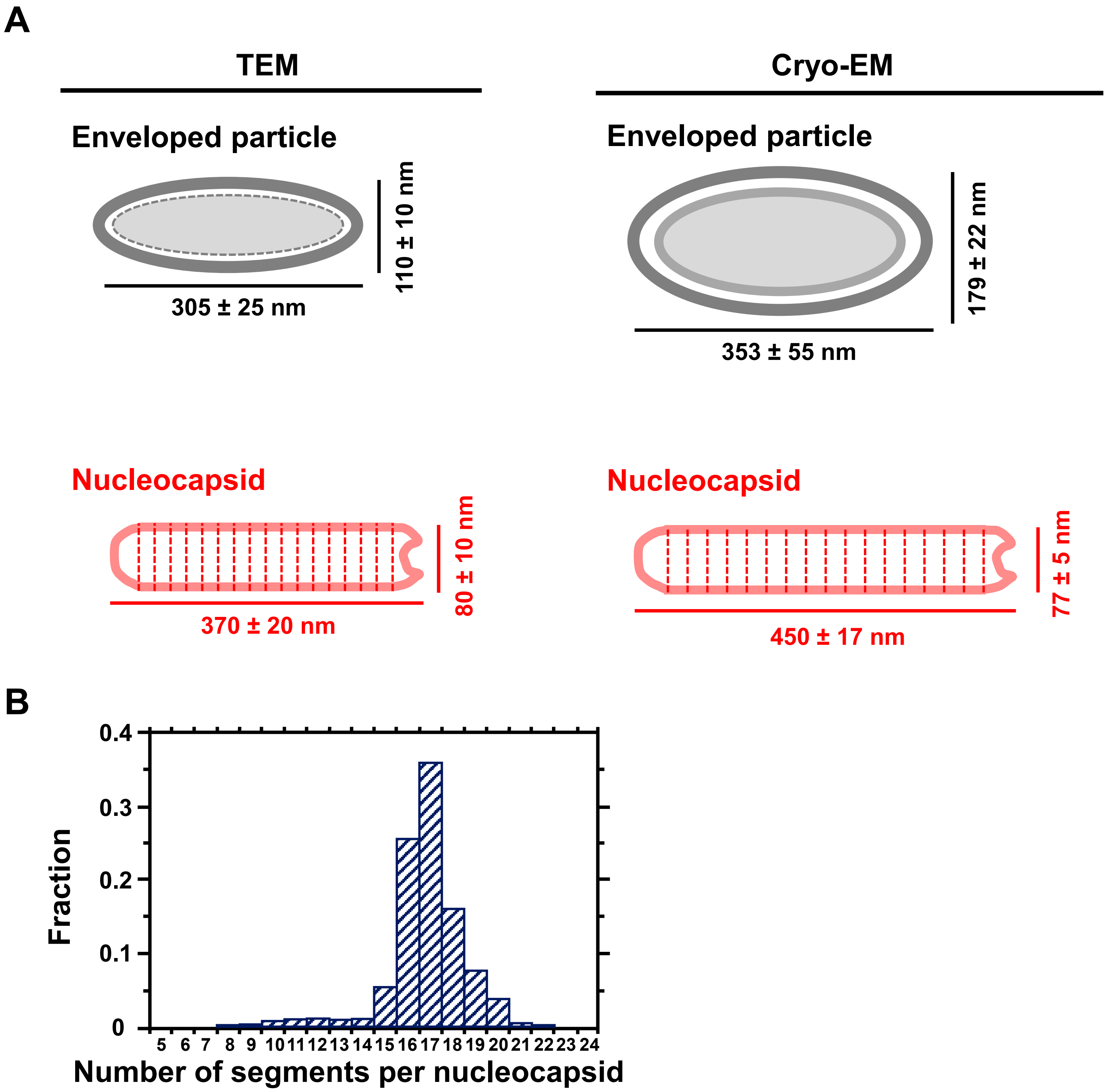
A
TEM
Enveloped particle
110 ± 10 nm
305 ± 25 nm
Cryo-EM
Enveloped particle
179 ± 22 nm
353 ± 55 nm
Nucleocapsid
80 ± 10 nm
370 ± 20 nm
Nucleocapsid
77 ± 5 nm
450 ± 17 nm
B
Fraction
0.4
0.3
0.2
0.1
0
5 6 7 8 9 10 11 12 13 14 15 16 17 18 19 20 21 22 23 24
Number of segments per nucleocapsid

Figure S1
